# Study on the Effect of Dexmedetomidine on Postoperative Cognitive Dysfunction and Inflammation in Aged Rats

**DOI:** 10.1155/2022/8830706

**Published:** 2022-07-01

**Authors:** Ming Hou, Qiongyu Zhang, Kai Deng

**Affiliations:** ^1^Department of Anesthesiology, The First Hospital Affiliated with Shandong First Medical University, Shandong, Jinan 250014, China; ^2^Department of Nephrology, Wenshang County People's Hospital,Shandong, Shandong, Jining 272599, China

## Abstract

**Objective:**

The aim of this study is to investigate the effect of dexmedetomidine on cognitive dysfunction and inflammatory cytokines in the hippocampus after surgery in aged rats.

**Methods:**

A total of 30 healthy male Sprague Dawley rats were divided into control group, sham group, and dexmedetomidine group. A splenectomy rat model was established and dexmedetomidine was intraperitoneally injected before operation. The cognitive function of rats was examined by Morris Water-Maze Test, open field experiment, and passive avoidance memory test. And the expression levels of IL-6, IL-1*β*, and TNF-*α* in the hippocampus were examined by ELISA.

**Results:**

The escape latency for 5 continuous days in dexmedetomidine group was significantly decreased comparing with control group (all *P* < 0.05). The number of times of swimming and the percentage of swimming time in dexmedetomidine group were significantly more than those in control group (all *P* < 0.05). What is more, rats in dexmedetomidine group had the decreased time of stay in the central square and the increased number of standing times in comparison with the control group, and the statistical differences were found (all *P* < 0.05). Compared with the control group, dexmedetomidine intraperitoneally injected before surgery could significantly inhibit the expression levels of IL-6, IL-1*β*, and TNF-*α* in the hippocampus, and there were statistical differences (all *P* < 0.05).

**Conclusion:**

Dexmedetomidine could significantly relieve the postoperative cognitive dysfunction in aged rats. The mechanism may be associated with the decreased inflammatory cytokines in the hippocampus.

## 1. Introduction

Postoperative cognitive dysfunction has been considered as a degenerative neurological disorder, which usually occurs in the elderly people. It is characterized by progressive deterioration of cognitive function and reduced self-card ability including personality changes, mental disorder, impairment of memory, and loss attention [1]. It was reported that among elderly patients with over 60 years old, the incidence rate of postoperative cognitive dysfunction after operation with anesthesia could reach 25% [2]. In recent years, with the increase of elderly population, postoperative cognitive dysfunction has become a main problem after operation. Some studies showed that the mechanism of postoperative cognitive dysfunction involved various factors such as dysfunction of central cholinergic system, inflammation, and apoptosis of nerve cells [3]. The exact mechanism remains unknown. Other studies revealed that inflammatory response played an important role in the pathogenesis of postoperative cognitive dysfunction [4]. The occurrence of neuroinflammation could be caused by the changes during operative procedures and anesthesia. It was confirmed that the increased levels of inflammatory cytokines could lead to the impairment of memory and learning [5]. Therefore, it is very meaningful and necessary to further explore the promising prevention and treatment strategy for postoperative cognitive dysfunction.

Dexmedetomidine, as an intravenous central sympatholytic drug, is a highly selective *α*-2 adrenergic receptor agonist. It is widely applied in the intensive care units and surgical rooms. Dexmedetomidine could produce analgesia, sedation, inhibition of the sympathetic activity, and antianxiety. Many studies showed that dexmedetomidine could reduce delirium in patients [6]. Some studies suggested that dexmedetomidine decreased the behavior of patients with postoperative cognitive dysfunction via protecting the function of nerve cells [7]. Other studies reported that dexmedetomidine did not affect the postoperative cognition in over 70-year old patients receiving elective operations [8]. Thus, further studies are required for evaluating the effects of dexmedetomidine on postoperative cognitive dysfunction and investigate the involved mechanisms [9].

The effects of dexmedetomidine on postoperative cognitive dysfunction and inflammatory cytokines in hippocampus of aged rats were investigated. Morris water maze test, open field test, and passive avoidance memory test were used to detect the cognitive function of rats. The results of this study will provide a theoretical basis for the clinical practice of elderly patients.

## 2. Material and Methods

### 2.1. Animals

This study was approved by the Animal Care and Use Committee of The First Hospital Affiliated with Shandong First Medical University and was conducted following the guidelines developed by the Chinese Association for Laboratory Animal Sciences. 30 male Sprague-Dawley (SD) aged rats, aged 18 months weighted 800–1000 g, were kept in Animal Centre of Shandong First Medical University. SD rats were housed with a 12 h light-dark cycle at room temperature 22 ± 1°C and a relative humidity of 45–75%. Food and water were freely available.

### 2.2. Construction of Animal Model

All the SD rats fasted for 12 h before the surgery. 2% sodium pentobarbital (50 mg/kg) was used for anesthetization. The surgical incision in the skin of SD rats was disinfected using iodophors. About 2 cm transverse incision was performed at the location of 1 cm under the lower edge of the left rib. The subcutaneous tissue was bluntly dissected layer by layer to enter into the abdominal cavity. Next, the vascular of spleen was ligated at the root and the spleen was resected. Finally, the abdomen was sutured using a 3–0 suture without bleeding. The whole surgery was finished aseptically. SD rats were back to the animal room for subsequent research after consciousness.

### 2.3. Animal Grouping

A total of 30 male SD rats were divided into the following three groups: sham surgery group, control group, and dexmedetomidine group. Rats in sham surgery group underwent operation without splenectomy, and 2 ml normal saline was intraperitoneally injected at the half an hour before operation. Rats in control group underwent splenectomy operation, and 2 ml normal saline was intraperitoneally injected at the half an hour before operation. Rats in dexmedetomidine group underwent splenectomy operation, and 20 *μ*g/kg of dexmedetomidine was intraperitoneally injected at the half an hour before operation.

### 2.4. Morris Water-Maze Test

The rats in each group were evaluated by the Morris water maze test including place navigation test and spatial probe test. The maze included a circular pool and a clear round platform. The pool was separated into four quadrants. The platform was placed in one quadrant below the water surface. Five days were taken for training cycles. And the rats from three groups were trained four times every day to observe the average daily escape latency. The interval time of each train was 120 s. Rats were released at different quadrants in the pool, facing toward the wall of the pool. The time for reaching the platform was recorded. When the rat did not arrive the platform within 60 s, it should be guided onto the platform and stayed for 10 s on the platform. The escape latency record of the rats was 60 s. After place navigation test, the platform was removed. The rats were trained one time each day for two days. Rats were placed at the same point of entry in the pool, facing toward the wall of the pool. The number of times of swimming for crossing the original platform was observed within 60 s. At the same time, the percentage of swimming time in the targeted quadrant in which original platform located was recorded.

### 2.5. Open Field Experiment

According to the previous studies, open field experiment was performed. Before experiment, 75% alcohol was used to clean the feces and urine of rats. The whole experiment time was 5 min. The light source was kept at the side of the box. Rats from three groups were placed in the open field test box. And the activities of rats were set as the observed index. It was recorded as one time positive activity when the forelegs were off of the ground.

### 2.6. Passive Avoidance Memory Test

According to the previous study, the shuttle box test was used to evaluate the passive avoidance memory test. The shuttle box was made of light and dark compartments. Each rat was kept in the light compartment with unconditioned stimulus for 5 s. The initiative avoiding latency was defined as the entering delay of each rat in the dark chamber. After 10 mins, the conditioned stimulus was conducted. And passive avoiding latency was considered as the delay of fleeing to safety. The times of avoiding were examined under the conditioned and unconditioned stimulus. The results of avoiding latency at 7th day were examined after training for six days.

### 2.7. Detection of Inflammatory Factors

Frozen hippocampus samples from three groups were obtained and were completely homogenized in Phosphate Buffer Saline. Then, the mixture was performed for centrifugation with 8000 × g for 15 mins under the conditions of 4°C. The supernatant was obtained and enzyme-linked immunosorbent assay (ELISA) was exploited for examining the expression levels of IL-6, IL-1*β*, and TNF-*α* in supernatant. The experimental procedure strictly followed the manufacturer's protocols.

### 2.8. Statistical Analysis

All data included in this study were performed using SPSS software version 23.0. The measurement data were expressed by Mean ± Standard Deviation (Mean ± SD); One-way ANOVA followed by post hoc bonferroni analysis was performed among the three groups. The enumeration data was presented as percentage or rate, and chi square test was applied for the comparison between two groups. *P* < 0.05 indicated significantly statistical differences.

## 3. Results

### 3.1. Comparison of Results of Morris Water-Maze Test

As shown in Table 1, in place navigation test, the difference in escape latency for 5 continuous days between control group and sham group had a statistical significance (all *P* < 0.05). Compared with the control group or sham group, the escape latency for 5 continuous days in dexmedetomidine group was significantly decreased, and there were obviously statistical differences (all *P* < 0.05).

As seen in Table 2, in terms of spatial probe test, there were significantly statistical differences for the number of times of swimming and the percentage of swimming time between control group and sham group (*P* < 0.05). The number of times of swimming and the percentage of swimming time in dexmedetomidine group were obviously more than those in control group or sham group, and the significant differences were found among the groups (*P* < 0.05).

### 3.2. Comparison of Results of Open Field Test

As shown in Table 3, the results of open field test revealed that splenectomy operation could obviously increase the time of stay in the central square and decrease the number of standing times in comparison with the control groups (all *P* < 0.05). In addition, the intraperitoneal injection of dexmedetomidine before splenectomy surgery could significantly decrease the time of stay in the central square and increase the number of standing times comparing with the control group (all *P* < 0.05).

### 3.3. Comparison of Results of Shuttle Box Test

The results of the shuttle box test were showed that rats in the control group have increased latency of the initiative avoiding (7.2 ±2.6 s vs 4.9 ±1.3 s, *P* < 0.05) and latency of the passive avoiding(12.6±1.9 s vs 10.1±1.8 s, *P* < 0.05) and reduced times of avoiding (5.7±1.5 s vs 11.7±2.1 s, *P* < 0.05) in comparison with the sham group. Moreover, rats in the dexmedetomidine group have decreased latency of the initiative avoiding (5.5±1.6 vs 7.2±2.6 s, *P* < 0.05) and latency of the passive avoiding (11.2±1.4 s vs 12.6±1.9 s, *P* < 0.05) and increased times of avoiding (8.5±1.8 vs 5.7±1.5, *P* < 0.05) comparing with the control group. The statistical differences were found among the groups, as seen in Table 4.

### 3.4. Comparison of the Expression Levels of Inflammatory Factors

ELISA analysis was used to detect the expression levels of inflammatory cytokines in the three groups. As shown in Table 5, compared with the sham group, the expression levels of IL-6, IL-1*β*, and TNF-*α* were significantly increased, and there were a statistical difference between two groups (all *P* < 0.05). Moreover, rats were intraperitoneally injected by dexmedetomidine in the dexmedetomidine group and had the decreased levels of IL-6, IL-1*β*, and TNF-*α* in the hippocampus in contrast to the control group. The significant differences were observed (all *P* < 0.05).

## 4. Discussion

Postoperative cognitive function is defined that there are no mental disorder in patients before operation and the impairments of orientation, memory and mental concentration appear in patients after operation. Previous studies showed that the incidence of postoperative cognitive function was 25.8% at one week after major noncardiac surgery in patients aged over 60 years old. At present, there is no effective treatment for postoperative cognitive function. Many studies showed that the reductions of neuron number, cognitive related neurotransmitters, and corresponding receptors could cause the decline of cognitive function [10, 11]. Other studies showed that surgical trauma was one of the high risk factors for the cognitive dysfunction [12]. This was because that surgical trauma could cause the release of inflammatory cytokines, which played an important role in the development of cognitive dysfunction [13]. To investigate whether surgical trauma could affect the cognitive function in rats, in this study, SD rats, aged 18 months, underwent splenectomy to simulate the conditions of postoperative cognitive dysfunction. In Morris water-maze test, shuttle box test, and open field test, the results revealed that compared with the sham group, the escape latency for 5 continuous days, the time of stay in the central square, the latency of the initiative avoiding, and the latency of the passive avoiding increased, while the number of times of swimming, the percentage of swimming time, the number of standing times, and the times of avoiding decreased in the control group. Thus, the adaptability, memory, learning, and cognitive abilities were damaged in these rats who received splenectomy. And the animal model of cognitive dysfunction was established successfully in the control group. In addition, the results of this study showed that the escape latency for 5 continuous days, the time of stay in the central square, the latency of the initiative avoiding, and the latency of the passive avoiding decreased, while the number of times of swimming, the percentage of swimming time, the number of standing times, and the times of avoiding increased in the dexmedetomidine group. Our study indicated that dexmedetomidine could relieve the cognitive dysfunction after surgery in aged rats. The results of this study were similar with previous studies [14].

Under the conditions of postoperative cognitive dysfunction, many immune cells and inflammatory cytokines in peripheral blood entered into the brain and acted in the central nerve system. It was reported that peripheral inflammatory mediators could activate the microglial cells to produce a lot of inflammatory factors [15]. Various inflammatory mediators such as TNF-*α*, IL-1*β*, and IL-6, in turn, could lead to a cascade of amplified inflammatory responses [16]. It was confirmed that excessive inflammation could irreversibly or reversibly damage the brain tissues and result in the impairment of cognitive function via different mechanisms such as necrosis and degeneration [17]. As we can see, hippocampus tissues in the brain are very sensitive to the increased inflammatory factors because of widely expressed receptors of inflammatory cytokines. Many studies suggested that the hippocampus was one of the most widely studied areas [18]. TNF-*α*, IL-1*β*, and IL-6, as a class of inflammatory factors, have a wide range of biological activities. Previous studies reported that these inflammatory mediators could lead to the edema of nerve cells and dysfunction of synaptic connections, finally resulting in cognitive dysfunction. It was reported that the elevated levels of IL-1*β* in the hippocampus tissues could cause the impairment of learning and memory functions [19]. Some studies reported that TNF-*α* and IL-1*β* could stimulate actin and played an important role in the process of neurodegenerative disease [20]. Hudetz et al. reported that the high level of IL-6 was correlated with short-term and middle-term memory after coronary artery bypass surgery [21]. Cibelli et al. reported that IL-6 could enhance the effects of IL-1*β* regulating the inflammatory response and cause the impaired hippocampus-dependent memory function [22]. In this study, IL-6, Il-1*β*, and TNF-*α* expression levels in the hippocampi tissues of rats were examined by ELISA. The results showed that these inflammatory factors were increased after splenectomy, which was in accordance with previous studies. Moreover, this study revealed that dexmedetomidine could obviously decrease the expression levels of IL-6, Il-1*β*, and TNF-*α*, indicating that inhibiting the expression levels of inflammatory factors in the brain served a role in improving postoperative cognitive function. Chen et al. reported that dexmedetomidine effectively improve the postoperative cognitive function in rats through inhibiting hippocampal inflammation induced by surgical trauma, which supported the hypothesis from our study [23].

There were some limitations in the present study. First, a single dose of dexmedetomidine was investigated in this study, so it is unknown whether the effects of dexmedetomidine on cognitive dysfunction are concentration dependent. Second, the effects of dexmedetomidine on a group of male rats were determined, and it is unclear whether the obvious differences for these effects were found between males and females. Finally, the observation and simple detection methods were exploited in this study, so further studies are required to explore the exact mechanism through which dexmedetomidine improves postoperative cognitive dysfunction and inhibit inflammation response.

## 5. Conclusion

This study showed that intraperitoneal injection of dexmedetomidine could significantly improve the cognitive dysfunction of aged rats after splenectomy. Morris water-maze test, open field test, and passive avoidance memory test were used to detect the cognitive function of rats. It was found that dexmedetomidine could significantly reduce postoperative cognitive dysfunction in aged rats. Therefore, this study will provide a potential strategy for the treatment of postoperative cognitive dysfunction in elderly patients.

## Figures and Tables

**Table 1:** Comparison of escape latency at different time points among the three groups (Seconds).

Groups	One day after surgery	Two days after surgery	Three days after surgery	Four days after surgery	Five days after surgery
Control group	60.4±2.5	51.4±2.2	46.6±1.9	35.7±1.3	31.5 ± 1.1
Sham group	54.3±1.5^*∗*^	36.6±1.1^*∗*^	23.8±0.8^*∗*^	15.1±0.6^*∗*^	11.4±0.5^*∗*^
Dexmedetomidine group	58.2±1.8^*∗*^^#^	42.7±2.8^*∗*^^#^	29.8±1.4^*∗*^^#^	24.2±1.1^*∗*^^#^	19.3±0.9^*∗*^^#^

*Note.* Compared with the control group, ^*∗*^*p* < 0.05; compared with the sham group, ^#^*P* < 0.05.

**Table 2:** Comparison of the number of times of swimming and the percentage of swimming time among the three groups.

Groups	Number of times of swimming (times/min)	Percentage of swimming time (%)
Control group	1.3±0.6	21.2±0.7
Sham group	3.5±0.9^*∗*^	36.9±1.2^*∗*^
Dexmedetomidine group	2.8±0.8^*∗*^^#^	32.4±0.9^*∗*^^#^

*Note.* Compared with the control group, ^*∗*^*p* < 0.05; compared with the sham group, ^#^*P* < 0.05.

**Table 3:** Comparison of results of the open field test among the three groups.

Groups	The time of stay in the central square (s)	Number of standing times (times)
Control group	8.2±1.6	7.1±0.9
Sham group	4.8±0.7^*∗*^	11.3±1.2^*∗*^
Dexmedetomidine group	6.4±1.1^*∗*^^#^	9.7±1.0^*∗*^^#^

*Note.* Compared with the control group, ^*∗*^*P* < 0.05; compared with the sham group, ^#^*P* < 0.05.

**Table 4:** Comparison of results of passive avoidance memory test among the three groups.

Groups	Latency of the initiative avoiding (s)	Latency of the passive avoiding (s)	Times of avoiding (times)
Control group	7.2±2.6	12.6±1.9	5.7±1.5
Sham group	4.9±1.3*∗*	10.1±1.8*∗*	11.7±2.1*∗*
Dexmedetomidine group	5.5±1.6*∗*#	11.2±1.4*∗*#	8.5±1.8*∗*#

*Note.* Compared with the control group, ^∗^*P* < 0.05; compared with the sham group, ^#^*P* < 0.05.

**Table 5:** Comparison of inflammatory cytokines among the three groups.

Groups	IL-6 (pg/mL)	IL-1*β* (pg/mL)	TNF-*α* (pg/mL)
Control group	55.8±8.5	124.6±26.8	40.2±7.1
Sham group	20.6±4.6*∗*	30.4±4.7*∗*	12.9±3.2*∗*
Dexmedetomidine group	40.5±6.1*∗*#	90.8±10.3*∗*#	27.5±6.8*∗*#

*Note.* Compared with the control group, ^∗^*P* < 0.05; compared with the sham group, ^#^*P* < 0.05.

## Data Availability

The experimental data used to support the findings of this study are available from the corresponding author upon request.
